# Studies on ICD guidelines, cardiac amyloidosis in HFpEF, and remote monitoring outcomes

**DOI:** 10.1007/s12471-025-01965-0

**Published:** 2025-05-22

**Authors:** Pim van der Harst

**Affiliations:** Department of Cardiology, Division of Heart and Lungs, University Medical Centre Utrecht, Utrecht, The Netherlands

This issue of the *Netherlands Heart Journal*, presents three original studies with valuable insights into evolving clinical practice in electrophysiology and heart failure care.

Evertz et al. report on the impact of the 2023 Dutch guideline for primary prevention ICD implantation in patients with non-ischaemic cardiomyopathy (NICMP) [1]. Their single-centre analysis shows the new criteria significantly reduce ICD implantations, but many ‘de-selected’ patients still received appropriate ICD therapies, highlighting the ongoing tension between patient selection and undertreatment, a challenge requiring further research.

Achten et al. present a systematic screening study of transthyretin amyloid cardiomyopathy (ATTR-CM) in an unselected heart failure with preserved ejection fraction (HFpEF) cohort [2]. They report a 3% prevalence, but find that one-third of diagnosed patients had no increased left ventricular wall thickness at diagnosis. These results highlight the risk of overreliance on wall thickness alone for ATTR-CM detection, emphasise the need for broader clinical awareness and possibly more refined screening strategies.

The HERO study by Adriaansen et al. evaluates remote monitoring (RM) in pacemaker patients at elevated stroke risk without prior atrial fibrillation [3]. Although the HERO registry shows high RM acceptance and substantial arrhythmia burden, it did not significantly improve time to arrhythmia detection vs. conventional care. These findings highlight considerations for RM integration into clinical workflows and suggest opportunities for smarter, more efficient remote care, potentially enhanced by artificial intelligence.

These studies highlight challenges and opportunities in cardiovascular care: refining indications using real-world data, detecting disease earlier, and meaningful use of technological innovations. Careful interpretation and focus on individual patient care remain essential as clinical practice evolves.

I hope you find these contributions both informative and inspiring for your practice.
